# Neuromuscular Load in Professional Women’s Handball: Segmentation of the Player Load and the Impacts at Group and Individual Level

**DOI:** 10.3390/s24175750

**Published:** 2024-09-04

**Authors:** Antonio Antúnez, Pablo López-Sierra, Helena Vila-Suárez, Sergio J. Ibáñez

**Affiliations:** 1 Grupo de Optimización del Entrenamiento y el Rendimiento Deportivo (GOERD), Universidad de Extremadura, 10003 Cáceres, Spain; antunez@unex.es (A.A.); sibanez@unex.es (S.J.I.); 2 Department of Sports’ Special Didactics, Universidade de Vigo, Campus A Xunqueira, S/N, 36005 Pontevedra, Spain; hvila33@gmail.com; 3 Grupo de Investigación en Ciencias del Deporte (INCIDE), Departamento de Educación Física y Deportiva, Universidade da Coruña, Oleiros, 15179 A Coruña, Spain

**Keywords:** vertical load, horizontal load, team sport, inertial devices, high performance

## Abstract

Handball is a team sport characterised by physical interaction with other opponents. This interaction produces a high load on the players that can manifest itself in various ways, from discomfort to prolonged injuries due to tears caused by excessive load. In order to establish correct protocols for application in women’s teams, context- and gender-specific reference data must be available. For this reason, the present research aims to find out how women’s teams in European competitions prepare for decisive matches during the match week, analysing the load in a segmented way and the level of specificity that should be achieved in training. Ex post facto research was used in which a total of 17 players belonging to a women’s first division handball team in Spain participated. The variables player load and impacts extracted from the use of Wimu ProTM inertial devices were analysed. The results showed a high neuromuscular load in players at this competitive level, especially in the variable impacts, reaching values per session of up to 1000 impacts. The individuality analyses show that the load varies significantly depending on the subject, which is why it is considered essential to establish protocols for strength work and load control in the most specific way possible.

## 1. Introduction

Handball is a high-intensity intermittent sport [1] in which players face during training and competition high efforts that involve a load whose evolution must be controlled and planned by the team’s coaching staff. The load planning will be conditioned by the sum of the objective internal load [2], subjective internal load [3], kinematic external load [4] and neuromuscular external load [5].

The neuromuscular load directly affects the performance and health of the players, as this type of load is generated by the execution of practical movements typical of sport in general and handball in particular, such as jumping, running, pivoting, throwing and defending [5]. This load is marked by the dynamic and frequent movements of handball, the numerous impacts that players receive, the numerous changes of direction, the implementation of tactical systems, etc., which can affect injuries caused by overload, such as stress fractures or tendinitis, underlining the importance of the physical-conditional development of players to control muscle fatigue and reduce the risk of injury [6].

The use of inertial devices is well established in various individual and team sports, with handball being one of the least active in the research field [7]. Taking into account that there are articles in handball that have demonstrated the validity of these devices for this sport both in laboratory tests and on the court [8,9], it is essential to investigate the physical load in handball in order to characterise training and competition and optimise the training and performance processes of the players.

The use of these devices and new technologies makes it possible to manage loads efficiently to try to reduce the risk of injury to players [6]. The possibility of collecting multiple variables by obtaining positioning data and accelerometers makes it possible to collect a lot of data with very high precision and frequency, obtaining information on many variables that must subsequently be discriminated. From the use of inertial devices in handball, data have been obtained on player positioning and play distribution [10], throwing speed in running and jumping [11] and even the differences in load in handball on the court and on the beach [12] or the physical load on handball referees [13]. Other studies have pointed out that decelerations could be related to injury risk due to overloading [14].

However, the use of reference values is not sufficient to control the load in team sports. One of the most complex situations for physical trainers in this type of sport is to combine group training with individualised training, as the loads in most situations must be specific to each player due to the heterogeneity between them [15]. Studies in the literature have shown that the load in handball is variable depending on the position and that individual values must be taken into account to improve performance [16] and reduce injury risk [17]. That is why it is necessary to know what types of load should be worked on an individual level and what type of load should be worked on a collective level in order to facilitate and make the training processes more efficient.

Nevertheless, studies in women’s handball using new technologies are very scarce and practically nonexistent in professional women’s handball. Values obtained in semi-professional women’s handball [18] show that players perform in competition with an average of 566.7 total accelerations per game and 419 decelerations. These, weighted to playing time, correspond to 19.06 accelerations per minute and 14.5 decelerations per minute. In order to find values relating to player load and impacts in professional women’s handball, it is necessary to consider studies in beach handball [19], where the physical load is far from that of professional handball. In these matches, the average number of impacts is 477.13, being 5.26 higher than 8G. In terms of player load, the values obtained are 14.35 a.u.

Understanding and controlling the neuromuscular load seems essential to manage the loads in professional handball and to achieve the maximum possible performance, as well as to ensure the health of the players. Therefore, the aim of this research was to analyse the objective neuromuscular load in women’s handball. In addition, the specific objectives were to find out the segmentation of the loads received by the players during training and to analyse the individuality of the different variables.

## 2. Materials and Methods

### 2.1. Design

The present research was classified as an ex post facto design, following the research methods proposed by O’Donoghue [20]. This is because the researchers do not intervene in the training processes, remaining on the sidelines during the session. It is the coaching staff who are in charge of task design and load planning. The researchers simply place the devices before training and remove them at the end of the training session, staying out of the way during the session, only supervising that everything works correctly by visualising the data in real time. The main focus of the research is then taken to the analysis of the data, giving that retrospective naturalisation of the interventions categorised as ex post facto. Due to this, it was a non-experimental research design, taking place in the natural context of sport, without the deliberate manipulation of training or variables during the research process [21].

### 2.2. Participants

The participants in this research were 17 professional female handball players (age = 25.53 ± 5.69 years; height = 168.35 ± 6.95 cm; weight = 67.88 ± 8.18 kg) belonging to the Spanish top division of women’s handball (Liga Guerreras) and the European league (EHF Euro Cup) during the 2022–2023 season. Of the 17 players on the roster, two were goalkeepers, six front lines, four wingers, and five pivots. A non-probabilistic convenience sample was used, as access to these data is very complicated due to the small population of professional athletes. Informed consent was given to all participants before starting the research, explaining the possible risks and benefits of participating in the study. The research was conducted following the criteria of the Declaration of Helsinki (2013) [22], the Ethical Standards in Sport and Exercise Science Research of Harriss et al. (2022) [23] and was approved by the University Bioethics Committee (233/2019). The investigation respected the framework of Organic Law 3/2018 of 5 December on Personal Data Protection and Guarantee of Digital Rights (2018) [24].

#### Eligibility Criteria

The following criteria were established for the selection of participants: (i) belonging to the team officially, (ii) having participated in at least 80% of the training sessions, and (iii) having been available for at least the last two matches.

Exclusion criteria were: (i) having had a lower body injury less than one month before the start of the measurement, (ii) having trained with lower body discomfort during one or more training sessions, and (iii) having been training in a national team during part of the data collection period.

### 2.3. Sample

Data were collected from all the training tasks of all the players who met the inclusion and exclusion criteria during one week of a competitive mesocycle in preparation for the regular league and European competition. For the statistical analysis, two databases were created, one for each dependent variable analysed. The total sample analysed is 142 cases collected in a total of 5 training sessions.

### 2.4. Variables

The independent variable of the study was the training sessions. The dependent variable was the neuromuscular load, specifically the Player Load (measurement based on the accumulation of accelerations in all axes of the plane) and the impacts received by the players measured in G forces. The variable player load corresponds to the vector sum of device accelerations in the 3-axes. The complete formula for its calculation can be found in the article by Reche-Soto et al. [25]. The variables were collected in global values, weighted by time and segmented by work zones. The variables were not manipulated by the researchers during data collection.

### 2.5. Instruments

**Figure 1:**
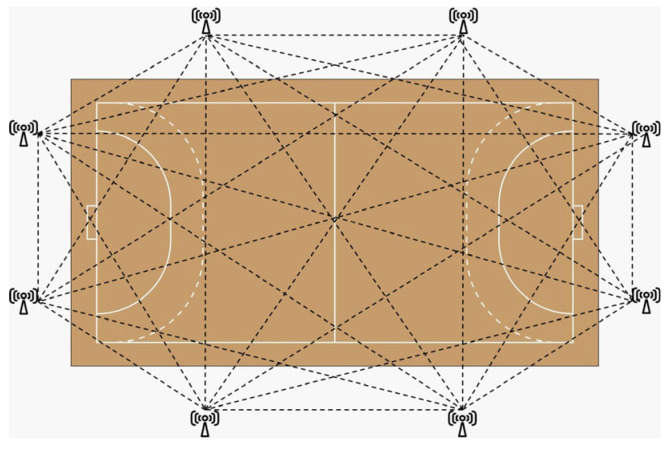
Configuration of the UWB system on the court.

The data were collected with Wimu ProTM devices (RealTrack Systems, Almeria, Spain). This required the installation of eight antennas around the field of play, establishing a signal system that allowed triangulating the position and movements of the players during the entire data collection of each session (Figure 1). The devices were positioned with a harness adjusted to the back, at the level of the T2–T4 thoracic vertebrae. These devices are valid and reliable for indoor interventions [26] and were used with 100 hz sampling. The devices included proprietary software for real-time data visualization and proprietary software for retrospective data processing.

### 2.6. Procedure

The physical trainers and coaching staff of the club were contacted in order to carry out the research. The players were informed of the benefits and disadvantages that could derive from participation in this research, the study being minimally invasive as the devices did not interfere with the players’ sporting practice. When all parties agreed, an informed consent form was drawn up and signed by all participants. This was followed by data collection.

During the training session, the data were monitored in real time through the software included with the inertial devices, *SVivoTM* version 923.4.0 (RealTrack Systems SL, Almeria, Spain, 2020). Once the training was over, the devices were removed and the data were saved in the cloud, stored there for further analysis.

The training sessions were carried out during the first five days of the week, with no rest in between. The sessions had an average duration of 67.4 min, with the longest session being the first one (79 min) and the shortest being the last one (60’). The total distance covered per session averaged 2.64 km per player, again with the longest distance covered being the first session (3.8 km) and the shortest being the last session (1.8 km). On a physical level, the first session was the one that generated the greatest load on the players, since it was the longest, the session in which the greatest distance was covered (and the greatest distance per minute as well), the one that reached the highest values of speed and acceleration, the greatest values of high intensity and player load (total and per minute) and the greatest total maximum impacts. The second session was the one that had more actions with very high G-force values and more intense falls. The third session was the one that generated the greatest imbalances in the right-left footprint. The fourth session was the one with the highest values in high intensity actions per minute and high impacts per minute. The last session was the softest with no maximum values.

Regarding methodological aspects, all sessions included unopposed situations and offensive and defensive Small-Sided Games. The last three also included Full Game situations. The first two sessions were the most balanced in both offensive and defensive aspects, with one unopposed task, two defensive and three offensive Small-Sided Games. The third session was eminently offensive. The fourth session was mainly tactical, with less effective playing time and one task of each type. Finally, the last session was low-load, with attacking and precision situations, and ended with a real situation that lasted half the session.

### 2.7. Statistical Analysis

A descriptive analysis of both variables was carried out first, in the case of the Player Load segmented by axes and in the case of the impacts segmented by G-force zones. Second, two linear mixed models were performed (one for the Player Load variable and the other for the impacts) to determine the possible differences in the variables, controlling the variance factor between subjects considering the evolution of the week. For this research, the default significance level was set at Alpha (α) = 5%. The level of statistical significance, *p*-value (*p*), was set at *p* = 0.05. Analyses were performed with the statistical programme JAMOVI (v2.3.28, The Jamovi Project, 2022).

## 3. Results

Table 1 shows the descriptive results of the Player Load variable and its different variables resulting from the segmentation by axes.

Table 1 shows that the players perform on average 0.67 au/min of Player Load, being mostly vertical and horizontal and in a smaller proportion of anteroposterior component.

Table 2 shows the mixed linear model to analyse the individuality of the variables, including only the total variables without weighting by time.

The results of the repeated measures linear mixed model showed a large improvement of the marginal R^2^ over the conditional R^2^ for all variables, controlling for the random factor of individual responses of subjects with a very high ICC (>0.70).

Table 3 shows the descriptive results of the variable impacts and their values divided by ranges.

It can be seen that horizontal impacts account for almost all the impacts received by the handball players. The number of impacts rises to values of over 10,000 impacts per session, with an average of 28 high intensity impacts and a maximum of 27 impacts received by a player in one session.

Table 4 shows the results of the linear mixed model used to analyse the individuality of the players in the different variables.

The results of the repeated measures linear mixed model showed a high improvement in the conditional R^2^ versus marginal R^2^ for the total impacts and horizontal impacts variables, finding an ICC > 0.70. The high impacts variables also show a significant improvement, especially for high total impacts, with an ICC above 0.05 for high total impacts and < 0.05 for high horizontal impacts.

## 4. Discussion

The aim of this study was to investigate the neuromuscular load in training handball players through the variables Player Load and Impacts in an elite women’s team. From the data, it is clear that the ability to accelerate, decelerate and change direction correctly is necessary for optimal physical performance in handball, which involves intense eccentric contractions that generate neuromuscular fatigue, which in turn leads to identifying the importance of being able to monitor the loads during training for greater control over possible injuries [14].

The descriptive results of the PlayerLoad variable in professional handball players show mean values of 0.67 au/min, reaching a maximum of 1 au/min. Research on handball players that includes the PlayerLoad variable is scarce and has been carried out in competition [27,28,29]. The studies indicated that Player Load values were similar for wingers, backs and pivots, except in the study by Wik, Luteberget and Spencer [29]. The main results indicate that elite women’s handball matches require high physical and physiological demands [28], but by using different devices, it is not possible to establish reference values for intensity and Player Load. Furthermore, different load responses have been recorded across matches, suggesting that coaches should be able to monitor match loads to be able to reproduce them in training in order to optimise the training load prescription according to the demand of each match. Another argument that highlights the importance of monitoring the external load is found in the complexity of controlling loads due to the long duration of the handball season, with the different performance objectives of each team and the possibility of qualifying and playing in international leagues, which conditions the work planning, making it a flexible process over time. To our knowledge, no previous research has focused on workload comparisons during a training week.

In the study presented by Font, Karcher, Reche, Carmona, Tremps and Irurtia [14], using a device similar to the one used in the present research, a season of an elite men’s team is monitored, where average Player Load values of 1.1 u.a. in matches throughout the season are shown, higher values than those presented during training by the players in this study. In the article by González-Haro et al. [30], the external load in amateur handball players was analysed, segmenting the load by specific positions. The Player Load values were around 0.59 and 0.83 on average, values that agree with those obtained in the present research. However, the difference between the samples is decisive for the interpretation of these data. The reference values for men’s handball are not applicable to women’s handball, since the loads borne by female players at the highest competitive level do not correspond to the resulting values for men’s handball at the same level [31].

Physical trainers should plan strength and conditioning training by including different types of exercises during training sessions to develop the ability of muscles and tendons to attenuate high eccentric forces, especially in players who tend to play on their backs because of their position and tactical tasks [32]. The assessment of these differences could represent crucial information for handball coaches and team practitioners in order to optimise the training load prescription according to the demand of each league match, both for specific off-court strength training as well as in specific on-court training designed by the coach.

Analysing the Player Load data obtained on the different axes, the similarity between the vertical and horizontal components stands out, as opposed to the differences between the two previous ones with the anteroposterior axis. Previous analyses of the segmentation of player load values according to acceleration axes are not known to exist in the literature. The findings of the present investigation reveal that vertical accelerations are practically the same as horizontal accelerations, so that the load generated by running and track displacements is equivalent to that generated by jumps and vertical oscillations in running. It is important to work on strength and power in all axes of the plane with movements that include free weights and different muscle groups [33], as the use of gym machines that work only one axis in the plane may not be optimal for sports performance in team sports [34].

The horizontal and total impacts received by the players are almost equal, as most of the impacts received by the players come from the interaction with other physical elements in the transverse plane. On average, the players receive more than 1000 impacts of a force between 0 and 4G per training session, but the values remain high until the high impact value (8G), where they are considerably reduced. The values collected in the study by González-Haro, Gómez-Carmona, Bastida-Castillo, Rojas-Valverde, Gómez-López and Pino-Ortega [30] are lower than those presented by the women’s team, finding mean values close to 60 impacts/min in the men’s amateur team and 80 impacts/min in the women’s senior team. These differences are probably due to the competitive level, as the players were training to enter the semi-final of a European competition. Other studies [35] have found differences in the impacts received by female players depending on the specific positions, with these differences being smaller as the hardness of the impacts increased. The intensity of the training sessions, especially with regard to the contact with teammates in the training sessions, influences the neuromuscular load that the players receive, so the loads must be adapted and controlled to the competitive level and players’ sex.

Analysis of individual players shows that the neuromuscular load varies greatly between subjects, hence the importance of player-specific load monitoring. The use of subjective tools to control the load for an entire team may not be sufficient in high performance, and other methods must be used to specifically assess the load that each player is able to withstand [36]. Studies such as that of González-Haro, Gómez-Carmona, Bastida-Castillo, Rojas-Valverde, Gómez-López and Pino-Ortega [30] found significant differences in the load depending on the specific positions, which could be a start to individualise in small work groups, but always trying to seek the greatest possible individualisation with the necessary technological support to do so.

Having objective information on the kinematic and neuromuscular external load demands of professional handball players will allow coaches at this level to design training programmes tailored to the specific demands of handball and its tactical game characteristics. These benchmark values are the first of their kind for this population of high-level professional players and should help to adjust training processes to match the demands of competition. However, PlayerLoad data should be interpreted and used with caution, and each brand uses a different algorithm to calculate this variable (some manufacturers calculate PlayerLoad from three-dimensional accelerometer data, while others use two-dimensional data for the calculation) [37].

The main limitation of this research is the transversality of the measurement, as a top-level team is measured in the most demanding training period of the season (preparation for a knockout semi-final match in European competition). However, this limitation is compensated for by the high quality of the data obtained, as it is a very representative week in terms of training and workload. As a prospective for the future, it is recommended that researchers carry out longer measurements and monitor competitions whenever possible.

## 5. Conclusions

Handball is a sport in which players bear high neuromuscular loads, essentially derived from the accumulation of accelerations in the horizontal and vertical planes and impacts in the horizontal axis. Strength work and training tasks should include complete movements in multiple planes, as well as work with free weights in the gym, combined with actions that involve a physical relationship with the environment.

## 6. Practical Applications

The results of this study underline the importance of monitoring training sessions to know the fatigue derived from accelerations and impacts in handball players, since they have a great importance when generating load in the players. This load should be taken into account to establish recovery and injury prevention plans during the season.

Due to the evidence of the high neuromuscular loads that players endure, it is recommended to perform off-court strength and power work based on multiaxis work with free weights and combining different muscle groups, reducing the work on a single plane of the gym machines. This off-court work should always be complemented with on-court work with tasks with many players in which there is continuous physical contact with teammates. Research should now focus on generating reference data in competition adapted to each level and sport context, paying special attention to impacts in the horizontal plane, in order to prepare players to withstand the eccentric load derived from the repetition of high G-force impacts throughout training or competition.

Finally, in the literature, consensus should be established on how to reference the load values derived from the Player Load with different devices, as the scarce evidence in this sport regarding this variable makes it difficult to apply reference values when using devices of different brands.

## Figures and Tables

**Table 1:** Descriptive results of the different variables related to the Player Load.

Variable	*N*	*X ± SD*	Minimum	Maximum
Player Load (a.u.)	70	48.37 ± 14.29	11.54	78.64
PL/min	70	0.67 ± 0.16	0.22	1.00
Horizontal Player Load (a.u.)	70	31.26 ± 9.21	7.69	49.29
hPL/min	70	0.43 ± 0.11	0.15	0.63
Vertical Player Load (a.u.)	70	31.55 ± 9.53	7.39	53.15
vPL/min	70	0.43 ± 0.11	0.14	0.67
Anteroposterior Player Load (a.u.)	70	19.37 ± 5.65	4.99	30.03
apPL/min	70	0.27 ± 0.06	0.10	0.38

**Table 2:** Linear mixed model of Player Load as a function of training session.

Variable	*Marginal R^2^*	*Conditional R^2^*	*AIC*	*ICC*	*p*
Player Load (a.u.)	0.34	0.85	492.44	0.78	<0.001
Horizontal Player Load (a.u.)	0.34	0.86	428.63	0.78	<0.001
Vertical Player Load (a.u.)	0.33	0.84	438.38	0.77	<0.001
Anteroposterior Player Load (a.u.)	0.37	0.83	367.38	0.72	<0.001

**Table 3:** Descriptive results of the variable impacts.

Variable	*N*	*X ± SD*	Minimum	Maximum
Total impacts (counter)	70	7196.91 ± 1757.88	2319	10,440
Total high impacts [>8G] (counter)	70	27.83 ± 19.48	4.00	87
Total impacts [0–2 G]	70	5829.71 ± 1360.27	2031	8347
Total impacts [2–4 G]	70	1002.09 ± 343.82	273	1789
Total impacts [4–6 G]	70	265.51 ± 120.24	10	567
Total impacts [6–8 G]	70	71.77 ± 37.41	1	178
Total impacts [8–10 G]	70	20.23 ± 14.44	3	67
Total impacts [>10 G]	70	7.60 ± 6.14	0	27
Horizontal impacts (counter)	70	7175.43 ± 2212.04	2211	11,308
High horizontal impacts [>8G] (counter)	70	3.50 ± 3.26	0	15
Horizontal impacts [0–2 G]	70	6646.17 ± 1995.46	2127	10,471
Horizontal impacts [2–4 G]	70	464.29 ± 204.71	79	970
Horizontal impacts [4–6 G]	70	52.09 ± 30.36	2	143
Horizontal impacts [6–8 G]	70	9.39 ± 6.76	0	30
Horizontal impacts [8–10 G]	70	2.19 ± 2.23	0	9
Horizontal impacts [>10 G]	70	1.31 ± 1.80	0	11

**Table 4:** Linear mixed model of impacts as a function of training session.

Variable	*Marginal R^2^*	*Conditional R^2^*	*AIC*	*ICC*	*p*
Total impacts (counter)	0.38	0.83	1171.38	0.73	<0.001
Total high impacts	0.12	0.68	581.43	0.64	<0.001
Horizontal impacts (counter)	0.35	0.84	1200.57	0.75	<0.001
High horizontal impacts	0.07	0.38	361.16	0.33	<0.146

## Data Availability

Data are unavailable due to ethical restrictions.
